# Public Health Professionals in Latin America Should Play a More Active Role in Tackling Drug Violence

**DOI:** 10.3389/ijph.2023.1605245

**Published:** 2023-05-10

**Authors:** Andrés Sánchez Pájaro

**Affiliations:** Center for Population Health Research, National Institute of Public Health, Cuernavaca, Mexico

**Keywords:** substance use, violence, public health workforce, Latin America, illicit drugs

**The IJPH series “Young Researcher Editorial” is a training project of the Swiss School of Public Health.**

Public health professionals in Latin America should expand research, social mobilization, policy development and implementation to tackle drug violence.

Despite an international ban on the production, sale, possession, and consumption of psychoactive substances (drugs) mandated by the 1961, 1971, and 1988 United Nations drug conventions, their unceasing demand in the United States (US) has been answered by a corresponding supply by drug-trafficking organizations in Latin America [1]. Though these international policies aim to protect people, they have precipitated irreparable harm to the Latin American population. As drug cartels diversify into other criminal activities, so has widespread devastating violence. This violence has become a key social issue in the region [2]: over the last 20 years, interpersonal violence became the second largest cause of disability-adjusted life years (DALYs) loss in the region (176,587,152 cumulative DALYs) [3]. Despite the continuing threat violence poses to public health, the response from public health professionals has been limited, rather, efforts to address this issue have mainly been assumed by actors in the criminal justice system and politics, with minor involvement from other fields [4].

Public health professionals typically work in governmental health departments, hospitals, universities, research centres or non-governmental organizations, carrying out one or more of the essential functions of public health. These include undertaking research and generating knowledge, developing, and implementing health policies, promoting legislation that protects the population’s health, incentivizing social participation and mobilization, and managing and promoting interventions to address the social determinants of health [5]. So far, most of the field’s efforts have been centred on reducing or treating drug use, including conducting research and implementing and evaluating prevention, treatment, rehabilitation and harm-reduction programs and strategies [6]. These actions have contributed to reducing drug use and its associated disease burden, but they are not sufficient [7].

Public health professionals must also engage in actions that reduce farming and drug production, processing, trafficking, and wholesale distribution. We could contribute to prevention in these earlier phases, before drugs reach consumers, and offer to help craft practical solutions, as public health professionals have done with tobacco products. A public health approach is broadly accepted and has been successful for regulating tobacco production (e.g., alternative crops financing), processing (e.g., legal regulation), wholesale (e.g., taxation) and retail (e.g., age restrictions, packaging) [8]. Because drug markets are transnational, international cooperation and shared aims must be the foundation for developing these effective solutions. Actions must be synchronized across countries; for example, interventions intended to reduce the effects of social determinants that lead people to farm and produce cocaine in Colombia and Peru (main producers), must be accompanied by actions that reduce the profitability of trafficking to the US (main consumer).

To decide which roles and actions would be appropriate for public health professionals in Latin America to assume, and how they can help prevent drug use at the pre-distribution level, we need to take four steps. First, we should generate robust evidence for new drug policies; for example, we might research alternative sources of income for coca farmers in Peru. Second, we should use this robust evidence to develop policies that will reduce drug use without increasing violence; for example, we might design a program that promotes alternate livelihoods to Peruvian coca farmers. Third, we should promote social participation and mobilization, and include key stakeholders so that new policies can be successfully implemented; for example, we can become advocates for the coca farmers program mentioned above. Fourth, we must counter the social determinants of drug use by coupling new policies to interventions that target underlying causes; for example, we could advocate implementing prevention strategies aimed at US cocaine consumers at the same time we intervene with Peruvian farmers. In conclusion, drug use is a multinational problem which has caused devastating violence in Latin America, and which should be more widely tackled by public health professionals through international cooperation, research, social mobilization, policy development and implementation.
